# Incidence of cytomegalovirus infection after kidney transplantation in the modern era of immunosuppression: the VINTAGE study

**DOI:** 10.1080/0886022X.2025.2491658

**Published:** 2025-04-22

**Authors:** Sumi Hidaka, Kazunari Tanabe, Shuzo Kobayashi

**Affiliations:** ^a^ Kidney Disease and Transplant Center, Shonan Kamakura General Hospital, Kanagawa, Japan; ^b^ Kidney Transplant and Robotic Surgery Center, Shonan Kamakura General Hospital, Kanagawa, Japan

**Keywords:** Kidney transplantation, cytomegalovirus, desensitization, diabetic nephropathy, older recipients

## Abstract

Cytomegalovirus (CMV) infection is a frequent complication following kidney transplantation that affects transplant outcomes. This study aimed to (i) estimate the 12-month cumulative incidence of CMV antigenemia (AG) in adult kidney transplant recipients not receiving antiviral prophylaxis, (ii) identify the risk factors for CMV AG, and (iii) assess the impact of CMV AG on transplant outcomes. This study included 128 living donor kidney recipients (aged ≥20 years) who underwent transplantation between 2012 and 2020. The mean recipient age was 52.8 ± 13.0 years. The overall positive CMV AG rates were 10.9%, 35.9%, 45.3%, 53.1%, and 59.4% (95% confidence interval (CI), 50.9–67.9) at 1, 2, 3, 6, and 12 months posttransplantation, respectively. The 12-month incidence rates in D−/R−, D−/R+, D+/R+, and D+/R − were 0%, 25.0%, 62.2%, and 81.3%, respectively. Multivariable analysis revealed that the risk of CMV AG increased with a stepwise increase in CMV serostatus risk category (hazard ratio (HR), 2.65; 95% CI, 1.66–4.21; *p* < .001) and recipient age (HR, 1.37 per 10-year increase; 95% CI, 1.14–1.65; *p* < .001). Positive CMV AG was associated with an increased risk of antibody-mediated rejection (HR, 21.40; 95% CI, 2.59–176.2; *p* = .005) and lower estimated glomerular filtration rate (*p* = .026). The risk of CMV AG is highest within the first 3 months posttransplant and persists for approximately 7–8 months in D + recipients. These findings underscore the importance of regular CMV monitoring for at least 6 months posttransplantation, particularly in centers employing preemptive therapy.

## Introduction

Cytomegalovirus (CMV) infection is the most common viral infection following kidney transplantation (KT) and can significantly affect transplant outcomes [1–4]. CMV may manifest as asymptomatic viremia or progress to mild-to-severe tissue-invasive disease [5].

Pretransplantation, anti-CMV IgG donor and recipient serostatus (D/R serostatus) consisting of a positive donor and a negative recipient (D+/R−) has been recognized as the highest risk factor for CMV infection/disease. The risk generally increases in the following order: D−/R−, D−/R+, D+/R+, and D+/R− [6,7].

Therefore, antiviral prophylaxis therapy is recommended by international guidelines and is widely accepted [8,9]. However, in Japanese individuals, the administration of valganciclovir (VGCV) or ganciclovir (GCV) can often induce severe thrombocytopenia and leukopenia [10], and was thus not recommended until 2023.

Modern immunosuppressive regimens have evolved to actively intervene in both cellular and humoral immunity, often by incorporating desensitization with anti-CD20 monoclonal antibodies in ABO-and/or HLA-incompatible donors [11–13]. Furthermore, the number of older KT recipients (KTRs) with diabetic nephropathy (DN) has increased [14].

While the qualitative risk order of CMV infection based on anti-CMV IgG D/R serostatus is well-known, the quantitative incidence of posttransplant CMV infection in the context of modern immunosuppressive therapies, particularly in older and DN KTRs, remains poorly investigated. Despite the well-known risk stratification, the lack of current incidence data has hindered the establishment of clear guidelines regarding the frequen posttransplant CMV infection monitoring. This knowledge gap may also challenge the design of future clinical studies aimed at optimizing the use of novel terminase and pUL97 kinase inhibitors in real-world clinical practice [15–19].

This study aimed to (i) estimate the cumulative incidence of CMV antigenemia (AG) over a 12-month period in adult KTRs who underwent living donor kidney transplantation (LDKT) without antiviral prophylaxis, (ii) identify independent risk factors for positive CMV AG, and (iii) determine the impact of positive CMV AG on subsequent transplant outcomes.

## Materials and methods

### Study population

This study included 128 consecutive adult KTRs aged ≥20 years who underwent LDKT between 2012 and 2020. These participants were selected from the ongoing Visualizing the Pathophysiology of Kidney Transplantation in Modern Age (VINTAGE) study, which is a hybrid retrospective and prospective observational cohort study [20,21]. The VINTAGE study was conducted at the Kidney Disease and Transplant Center and the Kidney Transplant and Robotic Surgery Center of the Shonan Kamakura General Hospital. The protocol used in the present study was approved by the relevant ethics committee (approval number TGE02305-024) and conducted in accordance with the Declaration of Helsinki and the Declaration of Istanbul on Organ Trafficking and Transplant Tourism. Informed consent was obtained through an opt-out procedure, with KTRs provided the opportunity to decline participation via an information sheet or hospital website.

### Immunosuppression and desensitization

The detailed immunosuppression and desensitization regimens have been reported previously [20]. Briefly, this regimen consisted of prolonged-release tacrolimus (PR-TAC), mycophenolate mofetil (MMF), and methylprednisolone (MP). Basiliximab was administered on the day of LDKT and on postoperative day 4. For KTRs with ABO and/or HLA incompatibility or sensitization due to a previous pregnancy, transplant, or transfusion, desensitization with rituximab (an anti-CD20 monoclonal antibody) and plasmapheresis was considered based on individual patient clinical assessments.

### Pretransplant CMV serostatus

Pretransplant anti-CMV IgG serostatus was determined using enzyme-linked immunosorbent assay (ELISA). Donor and recipient serostatus combinations were stratified into four groups: D−/R−, D−/R+, D+/R+, and D+/R−.

### Definition of CMV infection

CMV infection was defined as a positive CMV AG test result, irrespective of symptoms. CMV antigen-positive cells (polymorphonuclear leukocytes) in peripheral blood were detected using monoclonal antibodies against the CMV pp65 antigen (C10/C11 method, PRORAST^®^ CMV antigen kit). In the VINTAGE study, CMV infection data, including the date of diagnosis, were collected to track the potential progression from CMV infection to symptomatic and tissue-invasive CMV disease [5].

### Posttransplant CMV management

All KTRs, regardless of their CMV serostatus, underwent weekly CMV AG testing during hospitalization and at each outpatient visit after discharge. The CMV treatment strategy, once a patient tested positive for CMV AG, was as follows: In cases of R − serostatus before transplantation, the dosage of MMF was reduced, and KTRs were treated with VGCV (usual starting dosage 450–900 mg/d) to prevent progression to symptomatic CMV disease or tissue-invasive CMV disease, even if only one antigen-positive cell was detected. In cases of R + serostatus before transplantation, the dosage of MMF and PR-TAC was reduced based on the number of antigen-positive cells. However, if the number of positive cells reached approximately 5–10 positive cells per 100,000 white blood cells, antiviral treatment with VGCV was initiated (usual starting at a dose of 450–900 mg/d).

### Primary endpoint and follow-up

The primary endpoint was the time to first CMV AG positivity. The final follow-up date was 31 December 2021. No patients were lost to follow-up or withdrew consent. For KTRs with follow-up periods exceeding 12 months, the incidence of positive CMV results was censored at 12 months. Graft failures and rejections were also censored at 12 months.

### Estimated glomerular filtration rate

The estimated glomerular filtration rate (eGFR) was calculated using the following Japanese modified equation: 194 × (serum creatinine^−1.094^) × (age^−0.287^) × 0.739 (if female) [22].

### Statistical analysis

All statistical analyses were performed using SAS system version 9.4 TS1M7 (SAS Institute Inc., Cary, NC). Data are summarized as frequencies, mean ± standard deviation, or median (interquartile range [IQR]), as appropriate. Continuous variables with missing values were summarized using the available data. Fisher’s exact test was used for 2 × 2 contingency table analysis. Cumulative event rates were estimated using the Kaplan–Meier method, with a log-rank test for trend analysis. To assess the impact of the D/R serostatus on the time to first CMV AG positivity, a conventional Cox proportional hazards model was used. The proportional hazards assumption was confirmed using log–log survival plots. The effect of positive CMV AG results on subsequent rejection was evaluated using a time-dependent Cox model. A mixed-effects model was used to assess the effect of CMV AG status (positive vs. negative) on repeated eGFR measurements. Two-sided *p* values less than .05 were considered statistically significant. All analyses were performed at an independent data center (STATZ Institute Inc., Tokyo, Japan).

## Results

### Characteristics and immunosuppression

Table 1 summarizes the recipient, donor, and histocompatibility characteristics of the patients. The total pretransplant positivity rate for anti-CMV IgG was 82.8% in recipients and 89.1% in donors. D/R serostatus distribution was as follows: 16 D+/R− (12.5%), 98 D+/R+ (76.6%), 8 D−/R+ (6.3%), and 6 D−/R− (4.7%). The mean age of recipients was 52.8 ± 13.0 years, and the mean age of donors was 59.6 ± 10.3 years. The most common underlying disease was DN (28.9%). The study population included approximately 40% ABO-incompatible LDKTs. Table 2 shows the changes in PR-TAC trough levels, MMF dose, and MP dose. Most KTRs underwent rituximab (99.2%) or plasmapheresis (55.5%) therapy.

**Table 1.** Characteristics.

Variables	Value (*N* = 128)
Pretransplant CMV serostatus	
D+/R−	16 (12.5%)
D+/R+	98 (76.6%)
D−/R+	8 (6.3%)
D−/R−	6 (4.7%)
Recipient	
CMV seropositive	106 (82.8%)
Age (years)	52.8 ± 13.0
Men	83 (64.8%)
Duration of dialysis (months)^a^	8 [1, 25]
Preemptive kidney transplant	52 (40.6%)
Underlying diseases	
CGN	23 (18.0%)
IgAN	19 (14.8%)
ADPKD	20 (15.6%)
DN	37 (28.9%)
FSGS	4 (3.1%)
ANCA	5 (3.9%)
VUR	3 (2.3%)
Nephrosclerosis	7 (5.5%)
Others	10 (7.8%)
Donor	
CMV seropositive	114 (89.1%)
Age (years)	59.6 ± 10.3
Women	77 (60.2%)
Living donor	128 (100.0%)
Spousal donor	72 (56.3%)
Histocompatibility	
HLA mismatches	
A/B locus (0, 1, 2, 3, 4)	6/6/60/33/23
DR locus (0, 1, 2)	11/73/44
HLA-incompatible	9 (7.0%)
ABO-incompatible	50 (39.1%)

CMV: cytomegalovirus; D+: seropositive donor; D−: seronegative donor; R+: seropositive recipient; R−: seronegative recipient; CGN: chronic glomerulonephritis; DN: diabetic nephropathy; IgAN: immunoglobulin A nephropathy; ADPKD: autosomal dominant polycystic kidney disease; ANCA: anti-neutrophil cytoplasmic antibody; FSGS: focal segmental glomerulosclerosis; VUR: vesicoureteral reflux; HLA: human leukocyte antigen.

^a^ Median [interquartile range].

**Table 2.** Immunosuppression and desensitization.

Variables	Value (*N* = 128)
PR-tacrolimus	128 (100%)
Methylprednisolone	128 (100%)
Mycophenolate mofetil	128 (100%)
Basiliximab	128 (100%)
Desensitization	
Rituximab	127 (99.2%)
Plasmapheresis	71 (55.5%)
PR-TAC trough (ng/mL)	
2 weeks	9.1 ± 2.4
1 month	8.8 ± 2.6
3 months	7.5 ± 2.4
6 months	5.8 ± 2.0
12 months	5.1 ± 1.8
PR-TAC dose (mg/kg/day)	
2 weeks	0.14 ± 0.08
1 month	0.12 ± 0.06
3 months	0.10 ± 0.05
6 months	0.08 ± 0.04
12 months	0.07 ± 0.03
MMF dose (mg/day)	
2 weeks	1567 ± 267
1 month	1364 ± 331
3 months	1108 ± 389
6 months	970 ± 378
12 months	871 ± 393
MP dose (mg/day)	
2 weeks	8.1 ± 1.5
1 month	7.4 ± 1.3
3 months	5.6 ± 2.6
6 months	3.5 ± 1.4
12 months	3.2 ± 1.1

PR-TAC: prolonged-release tacrolimus; MMF: mycophenolate mofetil; MP: methylprednisolone.

Outpatient immunosuppressant medication dosages included four missing values but were summarized using available data.

### Overall positive CMV AG rate

**Figure 1.**
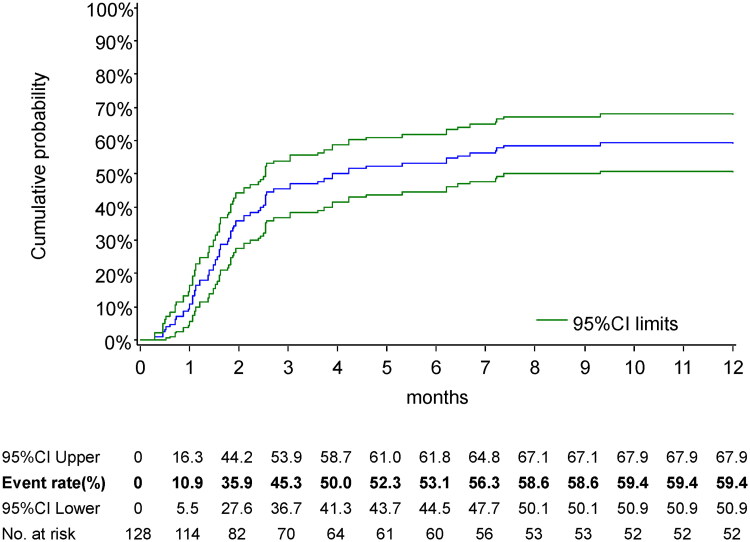
Kaplan–Meier’s estimates of time to first positive CMV antigenemia.

During the 12-month follow-up period, 76 of the 128 KTRs tested positive for CMV AG. Figure 1 shows the cumulative incidence rates of CMV AG positivity during the 12 months following LDKT. The rates were 10.9% (95% confidence interval (CI), 5.5–16.3), 35.9% (95% CI, 27.6–44.2), 45.3% (95% CI, 36.7–53.9), 53.1% (95% CI, 44.5–61.8), and 59.4% (95% CI, 50.9–67.9) at 1, 2, 3, 6, and 12 months, respectively.

### Positive CMV AG rate by D/R serostatus

**Figure 2.**
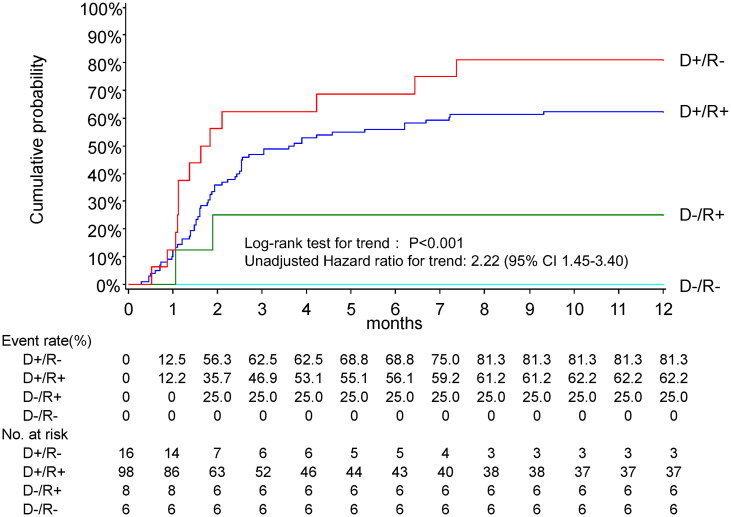
Kaplan–Meier’s estimates of time to first positive CMV antigenemia, stratified by D/R serostatus. Cl: confidence interval; D−: anti-CMV IgG-negative donor; R+: anti-CMV IgG-positive recipient; R−: anti-CMV IgG-negative recipient. One case each in the D+/R − and D+/R + groups progressed to tissue-invasive CMV disease (pneumonia).

Figure 2 illustrates the cumulative incidence of positive CMV AG according to the pretransplant D/R serostatus. The 3-month incidence rates in D−/R−, D−/R+, D+/R+, and D+/R − were 0%, 25.0%, 46.9%, and 62.5%, respectively. The 12-month incidence rates were 0%, 25.0%, 62.2%, and 81.3%, respectively. One case each in the D+/R − and D+/R + groups progressed to tissue-invasive CMV disease (pneumonia). Significant differences were observed among the four serostatus groups (log-rank test for trend: *χ*^2^ = 20.3, df = 1, *p* < .001), and the unadjusted hazard ratio (HR) for trend was 2.22 (95% CI, 1.45–3.40).

### Risk factors for positive CMV AG

We performed an exploratory analysis to identify factors associated with the development of CMV-positive CMV AG (Table 3). The risk of positive CMV AG increased with a stepwise increase in CMV serostatus risk category from D−/R − to D−/R+, D+/R+, and D+/R− (HR, 2.65; 95% CI, 1.66–4.21; *p* < .001). Recipient age was also identified as an independent risk factor, with an increased risk of CMV AG positivity per 10-year increase in age (HR, 1.37; 95% CI, 1.14–1.65; *p* < .001). These results were robust across different variable selection methods.

**Table 3.** Risk factors for positive CMV antigenemia.

Variables	Univariable			Multivariable (best subset selection method)		
	Crude HR	(95% CI)	*p* Value	Adjusted HR	(95% CI)	*p* Value
CMV serostatus^a^						
D−/R−, D−/R+, D+/R+, D+/R − order	2.22	(1.45–3.40)	<.001	2.65	(1.66–4.21)	<.001
Recipient age						
Per 10-year increase	1.27	(1.06–1.53)	.010	1.37	(1.14–1.65)	<.001
Donor age						
Per 10-year increase	1.34	(1.07–1.68)	.011			
Recipient sex						
Men	0.93	(0.59–1.49)	.771			
Donor sex						
Men	1.25	(0.80–1.97)	.331			
ABO compatibility						
Incompatible	1.50	(0.95–2.35)	.080			
HLA compatibility						
Incompatible	0.67	(0.25–1.84)	.438			
End-stage kidney disease						
Diabetic nephropathy	1.22	(0.75–1.98)	.421			
Rituximab^b^						
Yes		NA				
Pretransplant plasmapheresis						
Yes	0.75	(0.47–1.17)	.202			
PR-TAC trough^c^						
Per 1 ng/mL increase	0.95	(0.86–1.05)	.316			
MMF dose^c^						
Per 100 mg/day increase	1.01	(0.95–1.08)	.711			
MP dose^c^						
Per 1 mg/day increase	1.20	(1.04–1.39)	.016			

HR: hazard ratio; CI: confidence interval; PR-TAC: prolonged-release tacrolimus; MMF: mycophenolate mofetil; MP: methylprednisolone.

^a^ Hazard ratio was calculated based on CMV serostatus as a continuous value (D−/R− = 1, D−/R+ = 2, D+/R+ = 3, D+/R− = 4).

^b^ Hazard ratio could not be estimated because there were no events in the non-treated group.

^c^ Hazard ratios were calculated using repeated measures data (Table 2) as time-dependent covariates.

### Transplant outcomes by positive/negative CMV AG

Table 4 summarizes the incidence rates of graft failure (non-censored for death), biopsy-proven rejection, and eGFR according to CMV AG status (negative vs. positive). During the 12-month follow-up period, three KTRs (2.3%) in the positive CMV AG group experienced graft loss (*p* = .271). Positive CMV AG was associated with an increased risk of subsequent antibody-mediated rejection (ABMR) (HR, 21.40; 95% CI, 2.59–176.2; *p* = .005). To further explore this association, we performed a reverse analysis using ABMR as the independent variable and positive CMV-AG as the dependent variable. This analysis did not reach statistical significance (HR, 2.38; 95% CI, 0.96–5.90; *p* = .062). Throughout the follow-up period, eGFR was significantly lower in the positive CMV AG group compared to that in the negative group (*p* = .026). In the reverse analysis, a decrease in eGFR did not significantly increase the incidence of CMV AG positivity (*p* = .108).

**Table 4.** Transplant outcomes by negative/positive CMV antigenemia.

Endpoints	Negative CMV AG (*n* = 52)	Positive CMV AG (*n* = 76)	Analysis methods	
			Conventional	Reverse
			Explanatory variable: CMV AG	Explanatory variable: Rejection, eGFR
Graft failure^a^ (non-censored for death)	0 (0%)	3 (3.9%)	*p* = .271	–
Graft loss Acute TCMR		1 (1.3%)		
DWFG Portal vein thrombosis		1 (1.3%)		
Pneumonia		1 (1.3%)		
Biopsy-proven rejection^b^				
TCMR	1 (1.9%)	1 (1.3%)	*p* =.932	*p* = .988
ABMR	1 (1.9%)	9 (11.8%)	HR 21.40 (95% CI 2.59–176.2) *p* = .005	HR 2.38 (95% CI 0.96–5.90) *p* = .062
eGFR (mL/min/1.73 m^2^)^c^				
Pretransplant	8.9 ± 2.6	8.4 ± 2.5	*p* = .026	*p* = .108
2 weeks	46.1 ± 12.6	42.0 ± 15.2		
1 month	45.3 ± 12.3	41.0 ± 13.0		
3 months	43.9 ± 10.7	41.8 ± 11.1		
6 months	44.8 ± 11.8	40.6 ± 11.5		
12 months	45.1 ± 11.4	40.3 ± 11.5		

AG: antigenemia; DWFG: death with functioning graft; HR: hazard ratio; TCMR: T cell-mediated rejection; ABMR: antibody-mediated rejection; eGFR: estimated glomerular filtration rate.

The eGFR was calculated using the following equation: 194 × (serum creatinine^−1.094^) × (age^−0.287^) × 0.739 (if female) [22].

^a^ *p* Value was calculated using Fisher’s exact test.

^b^ *p* Values were calculated using the time-dependent Cox model.

^c^ *p* Value in the usual analysis was calculated using a mixed-effects model. The *p* value in the reverse analysis was calculated using the time-dependent Cox mode.

## Discussion

This study demonstrated that the overall 12-month cumulative incidence of positive CMV AG in adult LDKT recipients without antiviral prophylaxis was 59.4% (95% CI, 50.9–67.9%) (Figure 1). The risk of developing CMV AG was highest within the first 3 months but persisted for approximately 7–8 months post-LDKT, particularly in those with D+/R − and D+/R + serostatuses (Figure 2). CMV serostatus (with risks increasing from D−/R − to D−/R+, D+/R+, and D+/R−) and increasing recipient age were independently associated with the development of positive CMV AG (Table 3). Furthermore, a positive CMV AG was associated with a subsequent increase in ABMR and a decrease in eGFR (Table 4).

CMV infection has long been recognized as one of the most frequent complications of KT. Pretransplant assessment of anti-CMV IgG levels in both donors and recipients is recommended [1–3]. However, with the advancements in immunosuppressive regimens and antiviral therapies [2,3,23,24], patient demographics have evolved, with an increasing number of older and DN KTRs [25,26]. Additionally, CMV seropositive rates have been declining in Japan [27], and the proportion of D + R− serostatus has been increasing in the US across all organ transplants and is projected to continue rising [28]. Despite these trends, there has been a scarcity of published epidemiological data on the incidence of CMV infection/disease without antiviral prophylaxis since 2020, regardless of the diagnostic method used (AG assay or PCR) [29,30].

Consistent with previous reports [31], our analysis, which accounted for time dependence, identified CMV serostatus and recipient age as significant risk factors for the development of CMV-positive AG. Although donor age was a significant factor in the univariable analysis, it was not selected as an independent risk factor in the multivariable model. This can likely be attributed to collinearity with recipient age, as over half of the LDKTs were between spouses, and the selection of recipient age reflects the impact of immunosenescence associated with aging [32]. While an increase in the MP dose was also significant in the univariable analysis, this may be due to the suppression of tapering to compensate for reductions in the PR-TAC and MMF doses; however, the association between these two factors remains unclear.

Previous reports have presented conflicting findings regarding the temporal relationship between CMV infection and subsequent transplant outcomes, particularly rejection and graft function [33]. We hypothesized that this discrepancy may stem from challenges in handling time-dependent variables, as both CMV infection/disease and the outcomes of interest occur at irregular intervals after transplantation. To address this issue, we performed analyses that accounted for time dependence and reversed the explanatory and dependent variables. Our findings suggest that CMV AG can lead to subsequent ABMR, but not vice versa. In addition to its direct effects on infected organs, CMV can exert indirect effects, such as increasing susceptibility to other infections (e.g., through cytokine dysregulation) and elevating the risk of rejection by altering HLA expression [34]. Similarly, CMV AG appears to decrease subsequent eGFR. Notably, a decrease in eGFR does not appear to increase the risk of CMV AG.

This study has several limitations. Frist, the small sample size limited our ability to perform more detailed analyses, particularly regarding the impact of CMV serostatus and the effect of CMV AG on subsequent graft failure. Second, with regards to time-to-event analysis, this study only considered the first occurrence of positive CMV AG and does not account for subsequent infections or reactivation. Third, the observational design of the study means that residual confounding factors cannot be eliminated. Fourth, although PCR is widely used for CMV monitoring [35], it was not available for use our study because of its late introduction in Japan (after 2020). Fifth, the absence of a control group during the data collection and follow-up period resulted from the lack of health insurance coverage for CMV prophylaxis in Japan at that time, with preemptive therapy being the standard of care. In Japan, VGCV prophylaxis for KTRs was only covered by insurance from April 2023. Sixth, attributing CMV infection/disease solely to donor origin may be an oversimplification, as other sources of infection are possible [36,37]. Lastly, our findings have limited generalizability as they are based on a single-center study in Japan, where the prevalence of CMV IgG antibodies may differ from that in other countries [3,38].

This study has several strengths. First, it clarifies the order in which positive CMV AG, rejection, and graft function changes occur. Second, it reflects real-world clinical practice by including a population receiving modern immunosuppressive and desensitization therapies, with a substantial proportion of older and DN patients. Although posttransplant CMV management varies across transplant centers [2], this study provides valuable information that can inform the optimization of CMV management policies. Furthermore, our findings offer important implications for future clinical trials evaluating novel therapies, such as terminase and pUL97 kinase inhibitors.

In conclusion, the overall 12-month cumulative incidence of CMV AG in adult KTRs who underwent LDKT without antiviral prophylaxis was 59.4%. The risk of CMV AG is highest within the first 3 months posttransplant, and for D + recipients, this risk persists for approximately 7–8 months, regardless of the recipient’s initial serostatus. The CMV serostatus and increasing recipient age were independently associated with the development of CMV AG. Furthermore, CMV AG was associated with an increased risk of subsequent ABMR and decreased eGFR. These findings suggest that transplant centers with a policy of preemptive therapy for CMV should conduct regular monitoring for at least 6 months posttransplantation.

## Data Availability

The data supporting the findings of this study are available from the corresponding author upon reasonable request.

## Appendix

### The VINTAGE Investigators

#### Participating center

Kidney Disease and Transplant Center, Shonan Kamakura General Hospital, Kanagawa, Japan: Sumi Hidaka (principal investigator), Mizuki Yamano, Yasuhiro Mochida, Suguru Muraoka, Ayaka Mitomo, Haruka Maruyama, Kunihiro Ishioka, Machiko Oka, Hidekazu Moriya, Takayasu Ohtake, Yusuke Tsukamoto, Shuzo Kobayashi.

Kidney Transplant and Robotic Surgery Center, Shonan Kamakura General Hospital, Kanagawa, Japan: Keita Okubo, Kazunari Tanabe.

#### Transplant coordinator

Kidney Disease and Transplant Center, Shonan Kamakura General Hospital, Kanagawa, Japan: Midori Imai, Satomi Uchida.

#### Clinical research coordinator

Kidney Disease and Transplant Center, Shonan Kamakura General Hospital, Kanagawa, Japan: Midori Yonemura.

#### Biopsy evaluation center

Department of Diagnostic Pathology, Shonan Kamakura General Hospital, Kanagawa, Japan: Mitsuru Yanai.

#### Histocompatibility evaluation center

Regenerative Medicine, Shonan Research Institute of Innovative Medicine, Kanagawa, Japan: Tsutomu Sato.

#### Clinical drug information center

Kidney Disease and Transplant Center, Shonan Kamakura General Hospital, Kanagawa, Japan: Junko Horiuchi.

#### Biostatistics and data center

Department of Biostatistics, STATZ Institute Inc., Tokyo, Japan: Katsunori Shimada.

#### General management office

Kidney Disease and Transplant Center, Shonan Kamakura General Hospital, Kanagawa, Japan: Sumi Hidaka.
